# Differences in MEG gamma oscillatory power during performance of a prosaccade task in adolescents with FASD

**DOI:** 10.3389/fnhum.2013.00900

**Published:** 2013-12-25

**Authors:** Julia M. Stephen, Brian A. Coffman, David B. Stone, Piyadasa Kodituwakku

**Affiliations:** ^1^The Mind Research NetworkAlbuquerque, NM, USA; ^2^Department of Psychology, University of New MexicoAlbuquerque, NM, USA; ^3^Department of Pediatrics, University of New Mexico Health Sciences CenterAlbuquerque, NM, USA

**Keywords:** MEG, gamma-band, fetal alcohol spectrum disorders, adolescents, visual prosaccades

## Abstract

Fetal alcohol spectrum disorder (FASD) is characterized by a broad range of behavioral and cognitive deficits that impact the long-term quality of life for affected individuals. However, the underlying changes in brain structure and function associated with these cognitive impairments are not well-understood. Previous studies identified deficits in behavioral performance of prosaccade tasks in children with FASD. In this study, we investigated group differences in gamma oscillations during performance of a prosaccade task. We collected magnetoencephalography (MEG) data from 15 adolescents with FASD and 20 age-matched healthy controls (HC) with a mean age of 15.9 ± 0.4 years during performance of a prosaccade task. Eye movement was recorded and synchronized to the MEG data using an MEG compatible eye-tracker. The MEG data were analyzed relative to the onset of the visual saccade. Time-frequency analysis was performed using Fieldtrip with a focus on group differences in gamma-band oscillations. Following left target presentation, we identified four clusters over right frontal, right parietal, and left temporal/occipital cortex, with significantly different gamma-band (30–50 Hz) power between FASD and HC. Furthermore, visual M100 latencies described in Coffman etal. (2012) corresponded with increased gamma power over right central cortex in FASD only. Gamma-band differences were not identified for stimulus-averaged responses implying that these gamma-band differences were related to differences in saccade network functioning. These differences in gamma-band power may provide indications of atypical development of cortical networks in individuals with FASD.

## INTRODUCTION

Basic animal research and human behavioral and neuroimaging studies have contributed substantially to our understanding of the cortical networks involved in visual saccades (Goldberg et al., 2002; Pierrot-Deseilligny et al., 2002; Zhang and Barash, 2004; McDowell et al., 2008). Based on these and other studies, we now know that a complex network of cortical and subcortical regions interact to initiate saccades. These regions include subcortical structures such as superior colliculus, caudate nucleus of the striatum, thalamic nuclei, and cerebellum. The cortical network includes primary visual cortex, parietal eye fields, putatively located in medial intraparietal sulcus in humans, and supplementary and frontal eye fields (SEF and FEF; Clementz et al., 2001; Brown et al., 2006; Manoach et al., 2007; McDowell et al., 2008). These areas are differentially activated based on the nature of the saccade experiment: whether it involves prosaccades, including exogenous initiation of the visual saccade, or anti-saccades, where the response to the exogenous stimulus must be inhibited and an endogenous initiation of the saccade away from the target must be accomplished. Anti-saccade tasks invoke activation of additional executive control networks to inhibit the exogenous saccadic response. Further manipulations of the relative timing of the fixation and target stimuli can facilitate saccadic reaction times [SRT; e.g., providing a “gap” between the offset of the fixation and onset of the target stimulus – (Taylor et al., 1999; Dafoe et al., 2007)] and this additional time available for motor planning is associated with increased activity in FEF as demonstrated by functional magnetic resonance imaging (fMRI; Connolly et al., 2005). The exogenously initiated prosaccade task invokes the fronto-parietal saccadic network (e.g., Brown et al., 2006) and is less cognitively demanding than endogenous saccade tasks allowing investigators to assess the viability of the fronto-parietal saccade network in children.

Deficits in saccadic processing have been noted in multiple clinical populations including schizophrenia, attention deficit hyperactivity disorder and fetal alcohol spectrum disorders (FASDs; McDowell and Clementz, 2001; Manoach et al., 2002; Munoz et al., 2003; Feifel et al., 2004; Green et al., 2007). It has been proposed that visual saccades may provide a means to probe components of the cortical network underlying executive function and may provide an objective measure of impaired neural circuitry in these disorders of executive functioning (Manoach et al., 2002; Green et al., 2007). Visual prosaccade tasks provide an advantage over standard neuropsychological tests because a prosaccade task offers a measure of stimulus-initiated reflexive responses and hence is less susceptible to socio-cultural influences (Klein and Berg, 2001). This allows assessment of a broad spectrum of individuals with varying age, ability levels, and cultural backgrounds. Furthermore, there are numerous studies providing evidence of both gross and fine motor deficits in children with FASD (Bay and Kesmodel, 2010; Mattson et al., 2011; Simmons et al., 2012; Valenzuela et al., 2012). Therefore, a prosaccade task allows one to assess basic visual processing, visuo-motor integration abilities as well as deficits in motor execution in children with FASD.

Reynolds and colleagues (Green et al., 2007, 2009, 2013; Paolozza et al., 2013) identified deficits in both prosaccade and anti-saccade tasks in children with FASD relative to age-matched controls. These results provide evidence of delayed SRT (Green et al., 2007), differences in measures of fractional anisotropy within white matter tracts that correlate with SRT (Green et al., 2013), and larger variability in saccade accuracy in children with FASD relative to healthy controls (HC; Paolozza et al., 2013). Furthermore, a study in infant rats and mice demonstrated that the entire visual pathway, including retinal ganglion cells, subcortical structures and neurons in the visual cortex, is sensitive to ethanol with increased cell death following ethanol exposure (Tenkova et al., 2003). Our research group also identified a systematic delay in the onset of visual cortex activation (M100) in FASD relative to HC in response to both fixation (central) and target (peripheral) stimuli in a visual prosaccade task using magnetoencephalography (MEG) (Coffman et al., 2012). This is consistent with previous studies showing alterations in sensory processing in infants and children with FASD (auditory, somatosensory, and visual) in both animal and human studies (Medina et al., 2005; Church et al., 2012; Stephen et al., 2012).

Gamma oscillations in response to exogenous stimuli have been described in both animal and human studies (Tallon-Baudry et al., 1996; Uhlhaas and Singer, 2006). Since these initial studies were reported, it has become clear that gamma oscillations are integrally involved in the processing of both sensory and cognitive stimuli. In visual studies, gamma oscillations are implicated in feature binding across stimulus parameters, whereas cognitive studies suggest a role in working memory and higher cognitive functioning (Uhlhaas and Singer, 2006). However, the role of gamma oscillations in saccade processing is not well-understood (Van Der Werf et al., 2008, 2013). Based on animal models of FASD, prenatal alcohol exposure inhibits long-term potentiation (LTP) of GABA_A_ receptor-mediated postsynaptic potentials (Sanderson et al., 2009; Zucca and Valenzuela, 2010). Furthermore, the inhibitory signal provided by GABA_A_ modulates cortical oscillations (Hall et al., 2011). Based on these findings, we hypothesized that adolescents with FASD would show altered gamma modulations during performance of a prosaccade task. To test this hypothesis, we performed time-frequency analysis on the MEG dataset presented in Coffman et al. (2012). Our previous study focused on stimulus-averaged responses and did not characterize the broader cortical network associated with saccade execution; therefore, the current study focuses on the saccade-averaged response to understand the role of gamma oscillations in performing the saccade task.

## MATERIALS AND METHODS

### PARTICIPANTS

Forty-one adolescent participants (aged 12–21 years) were initially recruited. Participants or their parents (when children were under 18 years of age) completed the informed consent procedure prior to study participation in accordance with the Declaration of Helsinki. In this study we report on data from 35 adolescents from whom we obtained good-quality MEG data and successful prosaccade participation. Demographic characteristics of these participants are presented in **Table 1**.

**Table 1:** Participant demographics: mean (standard deviation).

	HC (*N* = 20)	FASD (*N* = 15)
Age (years)	16.3 (2.1)	15.3 (2.1)
IQ	108 (15)^*^	80 (15)^*^
Male/female (%male)	12/8 (60%)	10/5 (67%)
FASD sub diagnosis	–	8 FAS, 7 ARND

* *p* < 0.001.

Healthy control participants were included in the study if they attained an IQ score >70 and did not have any previous reports of neurodevelopmental disorders or known prenatal exposure to alcohol or other substances. Children were diagnosed as having fetal alcohol syndrome, partial fetal alcohol syndrome, or alcohol-related neurodevelopmental disorder using modified Institute of Medicine Criteria (Stratton et al., 1996) by a multidisciplinary team at the University of New Mexico Fetal Alcohol Diagnostic and Evaluation clinic. This clinical team was comprised of a developmental pediatrician, clinical neuropsychologist, and a child clinical psychologist. All children in the FASD group had confirmed prenatal alcohol exposure, which was established through several methods: (1) direct confirmation through the maternal interview; (2) eyewitness reports of drinking during pregnancy; (3) legal records confirming consumption of alcohol during pregnancy (e.g., DWI arrest); or (4) evidence of prenatal alcohol consumption in medical records. All participants completed the Wechsler Abbreviated Scale of Intelligence (WASI) to assess IQ and the Cambridge Gambling Task (CGT), from the Cambridge Neuropsychological Test Automated Battery (CANTAB), to assess executive function.

### PROCEDURES

**FIGURE 1:**
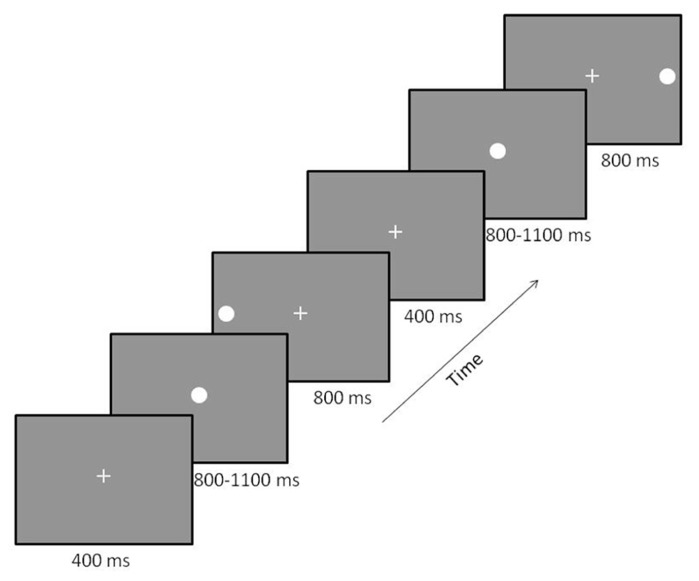
**Prosaccade paradigm.** Participants were presented with a fixation cross that was replaced by a fixation circle followed by either a left or right peripheral visual target. Two trials are displayed with a left followed by a right target. The order of the location of peripheral targets was randomized across trials to minimize anticipatory saccades.

Participants performed a prosaccade task (see **Figure 1**) described previously in Coffman et al. (2012). Briefly, participants sat in a reclining chair with their head in the MEG helmet. A back-projection screen was placed at a distance of 1 m from their nasion. The MEG-compatible SR Research Eyelink 1000 eye-tracker system was used to track eye-movement during the task. White visual stimuli were presented on a gray background using a Panasonic PT-D7700 DLP projector with a visual delay of 35.1 ± 0.2 ms. At the beginning of each trial, a small fixation cross was presented in central visual field. Participants were instructed to maintain fixation during this phase of the trial. Next, the fixation cross was replaced by a small white fixation circle (1°diameter, 50 cd/m^2^). This stimulus allowed the participant to prepare for the onset of the peripheral stimulus. To reduce anticipatory saccades, the peripheral stimulus (white circle – 1°diameter, 50 cd/m^2^) was presented after a variable delay (800–1100 ms). The peripheral stimulus was presented for 800 ms in either the left or right visual field at 15° eccentricity along the horizontal meridian. Left and right peripheral target stimuli were presented randomly with equal probability over 200 trials, providing 100 trials/condition. Participants were instructed to focus their gaze on the centrally and peripherally presented stimuli as quickly and accurately as possible. Once the peripheral target disappeared, the fixation cross reappeared to draw the participant’s gaze back to central fixation.

MEG data collection was performed using the Elekta Neuromag 306 channel Vectorview located within a magnetically shielded room. Prior to MEG data collection, standard bipolar electrocardiogram (ECG) and electrooculogram (EOG, horizontal and vertical) electrodes were placed to monitor heart rate, eye blinks, and eye movement for data quality purposes. ECG electrodes were placed bilaterally on left and right clavicles, and EOG electrodes were placed above and below the left eye (vertical EOG) and at the outer canthus of each eye (horizontal EOG). Four head position indicator (HPI) coils were placed around the hairline ensuring that the placement did not form a symmetric box pattern. All electrodes and coils were secured with tape. The position of the HPI coils and three fiducial points (left and right preauricular points and nasion) were recorded with the Polhemus 3D tracking device. Once the participant was comfortably seated in the MEG, the screen was positioned and the eye-tracker system was adjusted for the participant (infrared light source location and camera position were optimized to obtain good-quality pupil representation and corneal reflection). This was followed by a 9-point eye-tracker calibration sequence. Calibration was repeated until average eye location error between calibration and validation tests was less than 1° and maximum location error was less than 2° across all positions. Participants were given short (2–4 s) breaks every 10 trials throughout data collection to check calibration and to allow participants to rest their eyes. MEG data were collected at 1000 Hz with an online 0.01 high-pass filter and a 300 Hz anti-aliasing filter with head position monitored continuously throughout data collection.

### ANALYSIS

MEG data were preprocessed using Maxfilter. A default head position (default head center was based on the average head position of all 35 participants with good MEG data) was used along with the maxmove option of Maxfilter. This allowed for direct within-channel comparison of signals across participants and groups without concern of differences in head position within the MEG helmet during data collection. Trials were eliminated from further analysis in which eye position was not focused on the Cartesian coordinates of the central fixation point at the beginning of the saccade and the target at the end of the saccade, or direction of the first saccade greater than 30°/s following presentation of the peripheral visual stimulus was incorrect, as identified using the eye-tracker. That is, the participant was required to fixate on the central fixation point at the onset of the peripheral visual stimulus, saccade in the correct direction to the peripheral target, and reach the peripheral stimulus location following the saccade for each trial to be included in the time-frequency analysis.

Once preprocessing of the MEG data was complete, time-frequency analysis was performed to identify the temporal and spectral window of group differences in the gamma-band. Time-frequency analysis was performed using the tools available in the Fieldtrip toolbox (Oostenveld et al., 2011) and custom Matlab scripts. Morlet wavelets (width = 7 cycles) were used to develop time-frequency maps of activity across all trials within condition (fixation, left target, and right target). The time window of (-1000, 0) ms for each trial was analyzed for the left and right target, where 0 was the onset of the saccadic eye movement, as determined by the eye-tracker, and the time before zero denotes activity that initiates the saccadic response. The average spectral power for each frequency from the baseline time interval of (-500, -400) was removed from the rest of the time window. This time interval was prior to the onset of the visual stimulus for all participants, based on the longest SRT. The time-frequency maps were calculated for each trial individually and then averaged to provide a time-frequency map for each condition and participant at each sensor location. Only the time-frequency maps from the planar gradiometers are described further because planar gradiometers provide a measure of local brain activity since the maximal signal occurs directly over the source (Hamalainen et al., 1993). Furthermore, to facilitate interpretation, we combined the signals from the paired perpendicular planar gradiometers using the ft_combineplanar function in Fieldtrip. This reduced the number of sensors from 306 to 102 for further analysis.

To determine if differences in visual gamma activity influenced the saccadic network, we also performed time-frequency analysis on the stimulus-locked response for the left and right target conditions. The time (-100, 500 ms) and frequency (30–50 Hz) windows were analyzed with Morlet wavelets with 0 denoting the onset of the peripheral visual stimulus. The baseline time interval of (-100, 0 ms) was used for baseline correction of the time/frequency power in this analysis.

Once the time-frequency analysis was complete, we performed group comparisons using a two-stage approach to account for multiple comparisons as recommended by Fieldtrip developers (Maris and Oostenveld, 2007). The first stage identified time-frequency windows for which significant differences were identified by group. The time-frequency windows (contiguous regions within the 30–50 Hz range) within the (-400, 0) ms time window were compared statistically between groups for each channel. We chose an alpha (α) of 0.01 for each time-frequency point and required that at least 10 contiguous time-frequency points within the map reached the 0.01 significance threshold.

The second stage employed a permutation test for each time-frequency cluster identified in stage 1. The permutation analysis was performed within the clusters that were identified in the first stage. The participants were randomly reassigned group membership while maintaining the same percentage of HC and FASD participants. *T*-tests were applied to the identified clusters using the randomly reassigned group memberships. Reassignment was performed 200 times for each cluster and the absolute values of the *t*-statistic were calculated and summed across the cluster. If the *t*-statistics exceeded the summed absolute value from the original cluster in more than 10 permutations (5% threshold), the cluster was rejected. Regional group differences were identified when overlap of the time-frequency window of the significant clusters were identified in at least two adjacent channels.

Once significant group differences were identified and classified by regional cluster, the mean amplitude of the regional cluster, shared between sensors, was obtained for each participant to allow for comparisons of cluster gamma power with other behavioral measures. These comparisons were performed using Spearman’s correlation. Significance level was adjusted using Bonferroni corrections to account for the number of correlations performed. Finally, stepwise regression analysis was performed to test whether the visual latencies obtained in Coffman et al. (2012) predicted mean gamma power in any of the regional clusters.

## RESULTS

Of the 41 participants who were initially recruited, we were able to successfully track eye-movements in 35 of those individuals (20 HC and 15 FASD). As stated, trials were rejected for incorrect saccades or lack of compliance to the initial fixation point. On average there were 75 ± 3 trials per condition. There was a significant difference in the number of trials by group (*p* = 0.033), but this difference was not significant for left target alone (*p* > 0.05), which is the focus of our gamma frequency analysis. The mean age of the participants was not significantly different by group (*p* > 0.1). However, as expected, the FASD (IQ = 80) participants had a significantly lower IQ than HC (IQ = 108) participants (*p* < 0.01).

There were no significant differences in eye-tracking ability by group. The SRT and other saccade parameters are provided in **Table 2** along with *p*-values and effect sizes.

**Table 2:** Behavioral results prosaccade task: mean (standard deviation).

	HC (*N* = 20)	FASD (*N* = 15)	*p*-Value	Cohen’s d
Saccadic Reaction Times (SRT) (ms)	246 (19.5)	255 (27.8)	0.22	0.41
Right target SRT (ms)	243 (18.8)	253 (26.2)	0.19	0.45
Left target SRT (ms)	249 (25.8)	259 (31.9)	0.33	0.34
Percent correct	0.96 (0.02)	0.95 (0.04)	0.16	0.52
Saccade amplitude	12.4 (0.8)	12.0 (1.5)	0.35	0.32
Saccade peak velocity	335 (82.7)	317 (103.6)	0.57	0.20

**FIGURE 2:**
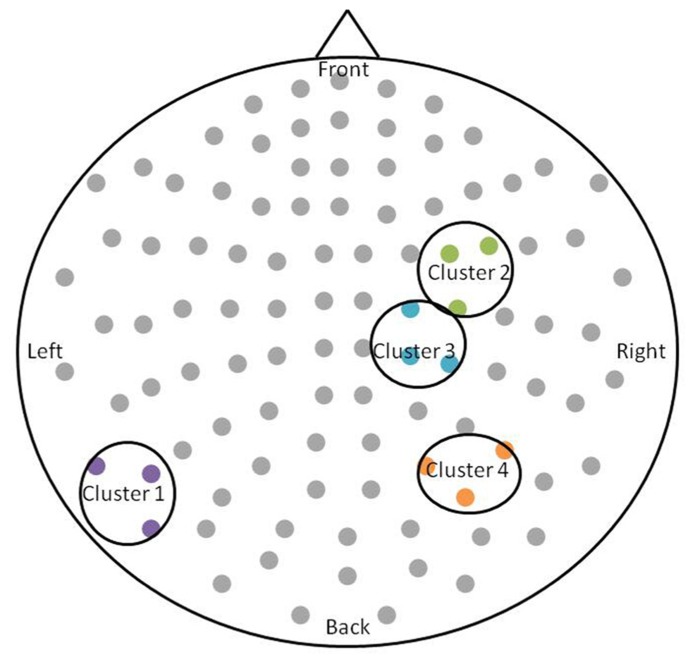
**Schematic of the significant clusters with respect to the MEG sensor array.** The sensor array is flattened and presented from a top-down view. Significant clusters are circled in black, and sensors within the clusters are colored. The significant clusters each included three channels.

**FIGURE 3:**
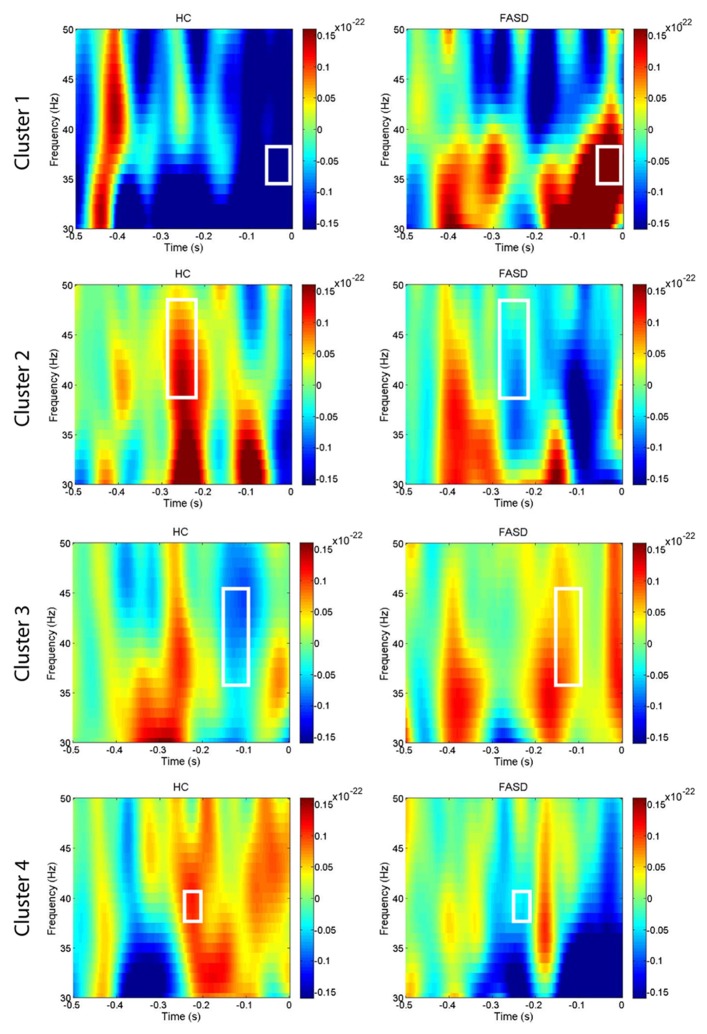
**Time-frequency plots.** The mean time-frequency plots for HC and FASD are shown from one representative channel from each of the four clusters. The time-frequency window with significant group differences in power is outlined by the white box. The cluster numbering is consistent with the locations shown in **Figure 2**.

**FIGURE 4:**
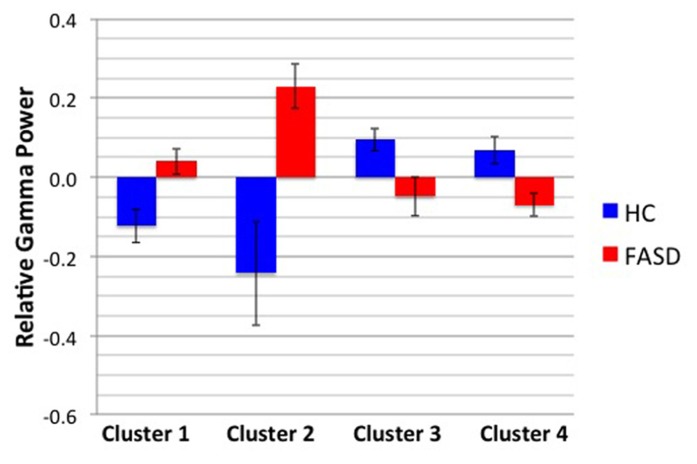
**Mean gamma power by cluster and group.** The mean power by group of the time-frequency window shown in **Figure 3** is displayed. Error bars denote standard error of the mean.

After permutation testing of the gamma-band clusters of the time-frequency maps for left and right targets, we only identified clusters that differed significantly by group in the left target condition in the saccade-averaged data. Four clusters were identified (location of these clusters relative to the sensor array is shown in **Figure 2**). Each cluster included three adjacent channels. Cluster 1 is located over the left occipital/temporal region. During the same data collection session, we also obtained somatosensory responses from a tactile stimulus. The initial somatosensory peak was localized in the channels in the vicinity of clusters 2 and 3, with cluster 2 focused slightly anterior to the somatosensory response and cluster 3 medial to the somatosensory response. This provides sufficient evidence that clusters 2 and 3 are located anterior to sensorimotor cortex, in the vicinity of FEF, and that cluster 4 is located over posterior parietal cortex. The time-frequency map from a representative channel for each cluster is presented in **Figure 3**. Finally, the mean power across the time-frequency windows that was identified to be significantly different by group is shown in **Figure 4**. No significant differences in gamma-band activity were identified for the stimulus-locked time-frequency analysis for either left or right target stimuli.

We performed correlations between the mean gamma cluster power and three measures: WASI IQ, prosaccade SRT, and CGT Impulsivity index (CGT group differences will be reported in a subsequent manuscript). None of these correlations reached statistical significance with Bonferroni correction. Finally, the results of the regression analyses to test whether the visual latencies obtained in Coffman et al. (2012) predicted gamma power in any of the four clusters are shown in **Table 3**. Target M100 latency positively predicted gamma amplitude in cluster 3 (located over right central cortex) in FASD only. There were no associations between gamma power and M100 latencies in HC.

**Table 3:** Linear regression of M100 latencies and mean cluster gamma amplitude.

Regressands	Regressors	β	Partial correlation	*R*^2^	*p*-Value
Cluster 1	*None*				
Cluster 2	*None*				
Cluster 3	Target M100 latency	0.621	0.44	0.38	0.024^*^
Cluster 4	*None*				

* *p* < 0.025 is significant accounting for testing across two groups.

## DISCUSSION

In summary, we identified group differences in gamma power in four time/frequency clusters located over different cortical regions in response to left target stimuli only. Furthermore, no differences in gamma-band power were identified for the stimulus-locked averages. These results indicate a hemispheric difference in saccadic processing in adolescents with FASD. Changes in gamma activity were not directly correlated with SRT; yet mean gamma amplitude of cluster 3, located over medial central regions consistent with SEF, was positively predicted by M100 latency to the peripheral target stimulus in FASD individuals only. These results provide evidence of altered gamma-band activity during saccade performance in FASD, a finding consistent with alterations in GABA_A_ in animal models of FASD.

As reported in our previous paper (Coffman et al., 2012), SRTs were not significantly different by group in this cohort. This differs from the previous results of Green et al. (2007), who reported group differences between children with FASD relative to HC. However, a more recent study (Paolozza et al., 2013) by the same group reported no significant difference in SRT in a different cohort of children with FASD. Despite the lack of difference in SRT, they confirmed that saccadic processing was still altered with reduced accuracy in saccade performance in children with FASD relative to age-matched HC. These differences across studies may represent variations in alcohol exposure patterns during the prenatal period within the FASD groups. Interestingly, gamma-band power did not correlate with SRT, yet gamma-band power in the medial FEF location (cluster 3) was positively predicted by M100 latency of the target stimulus in the FASD group only. In light of the early M100 deficits (Coffman et al., 2012), this increased gamma power indicates over-activation of gamma oscillations that may facilitate the saccadic response time. The mean M100 latency difference of 26 ms (Coffman et al., 2012) decreased to a mean SRT difference by group of 10 ms (**Table 2**). However, it is important to note that a significant correlation between M100 latency and SRT across groups was noted by Coffman et al. (2012); therefore increased gamma does not fully compensate for these early visual deficits. Furthermore, our analysis of the gamma-band power to the *stimulus-locked* response confirms that simple sensory differences are not driving the differences in gamma-band power in the saccadic *response-locked* activity. Additional studies are needed to further understand the link between stimulus-locked versus response-locked activity during saccade tasks.

The lack of a direct association between SRT and gamma-band activity indicates that performance of the visual saccade cannot be fully explained by gamma-band activity. A direct association between a behavioral outcome measure (e.g., SRT) and localized brain function would allow us to more directly understand the role of specific cortical activity. However, this association would more likely be identified if the analyses were performed on a trial-by-trial basis to allow us to view the variation in cortical activity that related to the same variation in individual trial SRTs. Yet, non-invasive methods do not provide a sufficient signal-to-noise ratio to perform this type of analysis for gamma-band oscillations. Recent results indicate that cross-frequency coupling links local with distributed activity and may explain the increased synchronization of gamma and concurrent desynchronization of alpha in posterior parietal cortex (Jensen and Colgin, 2007; Canolty and Knight, 2010). A broader view linking stimulus-locked and response-locked oscillatory activity may provide additional insights into how the brain performs visual saccades. Despite the lack of association between cortical activity and SRT, the reported differences in gamma power may provide a sensitive marker of prenatal alcohol exposure, independent of behavioral differences. The increase in gamma power in cluster 3 (right central – SEF) may represent compensatory activity, as increases in gamma-band power over contralateral parietal cortex corresponded to the planned saccade location identified by Van Der Werf et al. (2008). Despite this consistency in parietal activation, it should be noted that our *response-locked* results differ from the *stimulus-locked* increases in gamma power reported by Van Der Werf.

The locations of significant group differences are consistent with the prosaccade cortical network identified in previous saccade studies (Clementz et al., 2001; Pierrot-Deseilligny et al., 2002; Brown et al., 2006; Dyckman et al., 2007; McDowell et al., 2008) including occipital cortex, parietal cortex, and SEF and FEF. Furthermore, previous studies determined that activation in these regions is larger in the hemisphere contralateral to the target location (McDowell et al., 2005; Van Der Werf et al., 2008). Therefore, left target stimuli should preferentially activate regions in right hemisphere. This preference does not preclude activation of bilateral homologous regions, but the contralateral bias may provide a stronger signal-to-noise ratio that facilitates identification of group differences. The left occipital/temporal cluster (cluster 1) is not widely discussed as being a part of the saccade network, however, similar regions of activation were identified in a combined MEG/EEG prosaccade study (McDowell et al., 2005). Based on the location of cluster 2 relative to the somatosensory response, we propose that the differences in gamma-band power originate in right FEF. Although cluster 3 is immediately adjacent to cluster 2, the time-frequency windows do not overlap. The medial location of cluster 3 may denote supplementary eye field activity; however, other studies have reported both a medial and lateral region of FEF that are both activated by saccade tasks (McDowell et al., 2008). Employing source analysis of the time-frequency maps may help elucidate these adjacent, yet complementary group differences. Finally cluster 4 is located over parietal cortex, consistent with the intraparietal sulcus location of putative parietal eye fields.

Studies demonstrating deficits in right hemisphere connectivity in FASD may explain why group differences were found for left target but not right target stimuli. In addition to changes in the corpus callosum, Green et al. (2013) identified reduced FA in right inferior longitudinal fasciculus in FASD relative to HC. As an exploratory analysis, we changed the α from 0.01 to 0.05 in the stage 1 processing of the time-frequency maps for the right target to determine if differences were present with less stringent significance criteria. Two clusters remained significant after permutation testing and were measured over homologous left hemisphere regions as those identified in the left target condition. This provides evidence of consistent contralateral activation during a prosaccade task, but at the same time emphasizes that the right hemisphere effects are stronger than left hemisphere differences.

Although few studies have characterized gamma-band activity during saccade tasks, two previous MEG studies (Van Der Werf et al., 2008, 2013) examined alpha- and gamma-band activity concurrently in parietal cortex during the delay interval between the presentation of a peripheral stimulus and prior to a delayed saccade. Consistent with our current results gamma synchronization in parietal cortex was observed contralateral to the planned saccade. Van Der Werf et al. (2013) also determined that alpha desynchronization occurred in contralateral parietal cortex and was correlated with SRT. Interestingly, in the current results parietal gamma-band activity was decreased in FASD relative to HC, which may indicate impaired motor planning in FASD. However, it must be noted that the Van Der Werf study analyzed stimulus-locked rather than saccade-locked cortical responses. The consistency in parietal location may indicate that parietal cortex is involved in translation from stimulus-evoked responses to saccade-locked responses, but this cannot be directly tested using non-invasive methods. The delayed saccade design employed by Van Der Werf and colleagues may introduce additional frontal activations required to suppress the immediate saccade to the target stimulus, but Clementz et al. (2001) commented that the motor initiation network is consistent across simple (no-delay) and endogenously initiated (delay task) saccades. Our study which employed an experimental design to facilitate translation to younger children provides further evidence of the consistency of the nodes of the saccade network by identifying differences over regions widely reported in the saccade literature.

Based on animal studies (e.g., Zucca and Valenzuela, 2010), the excitatory/inhibitory balance in FASD individuals may be altered. These alterations may be manifested here as differences in gamma-band power in FASD relative to HC. Alterations in gamma-band power have been reported in other clinical disorders, including schizophrenia (Uhlhaas and Singer, 2006) and may be related to regional differences in GABA_A_ and altered inhibitory/excitatory ratios in neuropsychiatric disorders.

## CONCLUSION

This study provides an initial description of gamma-band differences between FASD and HC adolescents elicited by a prosaccade task. The deficits in right hemisphere are consistent with studies of other patient populations showing right hemisphere deficits in saccade tasks. The relationship between visual M100 latency and gamma power over right frontal regions may provide additional insights into the link between stimulus- and response-locked activity. Finally, this MEG measure provides higher sensitivity to group differences than behavioral SRTs alone and may be a useful marker of prenatal alcohol exposure in adolescents.

## Conflict of Interest Statement

The authors declare that the research was conducted in the absence of any commercial or financial relationships that could be construed as a potential conflict of interest.

## References

[B1] Bay B. Kesmodel U. S. (2010). Prenatal alcohol exposure - a systematic review of the effects on child motor function. *Acta Obstet. Gynecol. Scand.* 90 210–226 10.1111/j.1600-0412.2010.01039.x 21306306

[B2] Brown M. R. Goltz H. C. Vilis T. Ford K. A. Everling S. (2006). Inhibition and generation of saccades: rapid event-related fMRI of prosaccades, antisaccades, and nogo trials. *Neuroimage* 33 644–659 10.1016/j.neuroimage.2006.07.002 16949303

[B3] Canolty R. T. Knight R. T. (2010). The functional role of cross-frequency coupling. *Trends Cogn. Sci.* 14 506–515 10.1016/j.tics.2010.09.001 20932795 PMC3359652

[B4] Church M. W. Hotra J. W. Holmes P. A. Anumba J. I. Jackson D. A. Adams B. R. (2012). Auditory brainstem response (ABR) abnormalities across the life span of rats prenatally exposed to alcohol. *Alcohol. Clin. Exp. Res.* 36 83–96 10.1111/j.1530-0277.2011.01594.x 21815896 PMC3210930

[B5] Clementz B. A. Mcdowell J. E. Stewart S. E. (2001). Timing and magnitude of frontal activity differentiates refixation and anti-saccade performance. *Neuroreport* 12 1863–1868 10.1097/00001756-200107030-00020 11435913

[B6] Coffman B. A. Kodituwakku P. Kodituwakku E. L. Romero L. Sharadamma N. M. Stone D. (2012). Primary visual response (M100) delays in adolescents with FASD as measured with MEG. *Hum. Brain Mapp.* 34 2852–2862 10.1002/hbm.22110 22674650 PMC3993092

[B7] Connolly J. D. Goodale M. A. Goltz H. C. Munoz D. P. (2005). fMRI activation in the human frontal eye field is correlated with saccadic reaction time. *J. Neurophysiol.* 94 605–611 10.1152/jn.00830.2004 15590732

[B8] Dafoe J. M. Armstrong I. T. Munoz D. P. (2007). The influence of stimulus direction and eccentricity on pro- and anti-saccades in humans. *Exp. Brain Res.* 179 563–570 10.1007/s00221-006-0817-8 17171535

[B9] DyckmanK. A.CamchongJ.ClementzB. A.McdowellJ. E. (2007). An effect of context on saccade-related behavior and brain activity. *Neuroimage* 36 774–784 10.1016/j.neuroimage.2007.03.02317478104

[B10] Feifel D. Farber R. H. Clementz B. A. Perry W. Anllo-Vento L. (2004). Inhibitory deficits in ocular motor behavior in adults with attention-deficit/hyperactivity disorder. *Biol. Psychiatry* 56 333–339 10.1016/j.biopsych.2004.06.019 15336515

[B11] Goldberg M. E. Bisley J. Powell K. D. Gottlieb J. Kusunoki M. (2002). The role of the lateral intraparietal area of the monkey in the generation of saccades and visuospatial attention. *Ann. N. Y. Acad. Sci.* 956 205–215 10.1111/j.1749-6632.2002.tb02820.x 11960805

[B12] Green C. R. Lebel C. Rasmussen C. Beaulieu C. Reynolds J. N. (2013). Diffusion tensor imaging correlates of saccadic reaction time in children with fetal alcohol spectrum disorder. *Alcohol. Clin. Exp. Res.* 37 1499–1507 10.1111/acer.12132 23551175

[B13] Green C. R. Mihic A. M. Brien D. C. Armstrong I. T. Nikkel S. M. Stade B. C. (2009). Oculomotor control in children with fetal alcohol spectrum disorders assessed using a mobile eye-tracking laboratory. *Eur. J. Neurosci.* 29 1302–1309 10.1111/j.1460-9568.2009.06668.x 19302166

[B14] Green C. R. Munoz D. P. Nikkel S. M. Reynolds J. N. (2007). Deficits in eye movement control in children with fetal alcohol spectrum disorders. *Alcohol. Clin. Exp. Res.* 31 500–511 10.1111/j.1530-0277.2006.00335.x 17295736

[B15] Hall S. D. Stanford I. M. Yamawaki N. Mcallister C. J. Ronnqvist K. C. Woodhall G. L. (2011). The role of GABAergic modulation in motor function related neuronal network activity. *Neuroimage* 56 1506–1510 10.1016/j.neuroimage.2011.02.025 21320607

[B16] Hamalainen M. Hari R. Ilmoniemi R. J. Knuutila J. Lounasmaa O. V. (1993). Magnetoencephalography - theory, instrumentation, and applications to noninvasive studies of the working human brain. *Rev. Mod. Phys.* 65 413–497 10.1103/RevModPhys.65.413

[B17] Jensen O. Colgin L. L. (2007). Cross-frequency coupling between neuronal oscillations. *Trends Cogn. Sci.* 11 267–269 10.1016/j.tics.2007.05.003 17548233

[B18] Klein C. Berg P. (2001). Four-week test-retest stability of individual differences in the saccadic CNV, two saccadic task parameters, and selected neuropsychological tests. *Psychophysiology* 38 704–711 10.1111/1469-8986.3840704 11446584

[B19] Manoach D. S. Lindgren K. A. Cherkasova M. V. Goff D. C. Halpern E. F. Intriligator J. (2002). Schizophrenic subjects show deficient inhibition but intact task switching on saccadic tasks. *Biol. Psychiatry* 51 816–826 10.1016/S0006-3223(01)01356-7 12007456

[B20] Manoach D. S. Thakkar K. N. Cain M. S. Polli F. E. Edelman J. A. Fischl B. (2007). Neural activity is modulated by trial history: a functional magnetic resonance imaging study of the effects of a previous antisaccade. *J. Neurosci.* 27 1791–1798 10.1523/JNEUROSCI.3662-06.2007 17301186 PMC6673726

[B21] Maris E. Oostenveld R. (2007). Nonparametric statistical testing of EEG- and MEG-data. *J. Neurosci. Methods* 164 177–190 10.1016/j.jneumeth.2007.03.024 17517438

[B22] Mattson S. N. Crocker N. Nguyen T. T. (2011). Fetal alcohol spectrum disorders: neuropsychological and behavioral features. *Neuropsychol. Rev.* 21 81–101 10.1007/s11065-011-9167-9 21503685 PMC3410672

[B23] McDowell J. E. Clementz B. A. (2001). Behavioral and brain imaging studies of saccadic performance in schizophrenia. *Biol. Psychol.* 57 5–22 10.1016/S0301-0511(01)00087-4 11454432

[B24] McDowell J. E. Dyckman K. A. Austin B. P. Clementz B. A. (2008). Neurophysiology and neuroanatomy of reflexive and volitional saccades: evidence from studies of humans. *Brain Cogn.* 68 255–270 10.1016/j.bandc.2008.08.016 18835656 PMC2614688

[B25] McDowell J. E. Kissler J. M. Berg P. Dyckman K. A. Gao Y. Rockstroh B. (2005). Electroencephalography/magnetoencephalography study of cortical activities preceding prosaccades and antisaccades. *Neuroreport* 16 663–668 10.1097/00001756-200505120-00002 15858402

[B26] Medina A. E. Krahe T. E. Ramoa A. S. (2005). Early alcohol exposure induces persistent alteration of cortical columnar organization and reduced orientation selectivity in the visual cortex. *J. Neurophysiol.* 93 1317–1325 10.1152/jn.00714.2004 15483067

[B27] Munoz D. P. Armstrong I. T. Hampton K. A. Moore K. D. (2003). Altered control of visual fixation and saccadic eye movements in attention-deficit hyperactivity disorder. *J. Neurophysiol.* 90 503–514 10.1152/jn.00192.200300192.2003 12672781

[B28] Oostenveld R. Fries P. Maris E. Schoffelen J. M. (2011). FieldTrip: open source software for advanced analysis of MEG, EEG, and invasive electrophysiological data. *Comput. Intell. Neurosci.* 2011 156869 10.1155/2011/156869 PMC3021840 21253357

[B29] Paolozza A. Titman R. Brien D. Munoz D. P. Reynolds J. N. (2013). Altered accuracy of saccadic eye movements in children with fetal alcohol spectrum disorder. *Alcohol. Clin. Exp. Res.* 37 1491–1498 10.1111/acer.12119 23578065

[B30] Pierrot-Deseilligny C. Ploner C. J. Muri R. M. Gaymard B. Rivaud-Pechoux S. (2002). Effects of cortical lesions on saccadic: eye movements in humans. *Ann. N. Y. Acad. Sci.* 956 216–229 10.1111/j.1749-6632.2002.tb02821.x 11960806

[B31] Sanderson J. L. Donald Partridge L. Valenzuela C. F. (2009). Modulation of GABAergic and glutamatergic transmission by ethanol in the developing neocortex: an in vitro test of the excessive inhibition hypothesis of fetal alcohol spectrum disorder. *Neuropharmacology* 56 541–555 10.1016/j.neuropharm.2008.10.012 19027758 PMC2910524

[B32] Simmons R. W. Nguyen T. T. Levy S. S. Thomas J. D. Mattson S. N. Riley E. P. (2012). Children with heavy prenatal alcohol exposure exhibit deficits when regulating isometric force. *Alcohol. Clin. Exp. Res.* 36 302–309 10.1111/j.1530-0277.2011.01625.x 22014260 PMC3578740

[B33] Stephen J. Kodituwakku P. Kodituwakku E. L. Romero L. Peters A. M. Sharadamma N. M. (2012). Delays in auditory processing identified in preschool children with FASD. *Alcohol. Clin. Exp. Res.* 36 1720–1727 10.1111/j.1530-0277.2012.01769.x 22458372 PMC3390452

[B34] Stratton K. Howe C. Battaglia F. P Institute of Medicine. (1996). *Fetal Alcohol Syndrome: Diagnosis, Epidemiology, Prevention, and Treatment*. Washington: National Academy Press

[B35] Tallon-Baudry C. Bertrand O. Delpuech C. Pernier J. (1996). Stimulus specificity of phase-locked and non-phase-locked 40 Hz visual responses in human. *J. Neurosci.* 16 4240–4249 8753885 10.1523/JNEUROSCI.16-13-04240.1996 PMC6579008

[B36] Taylor T. L. Klein R. M. Munoz D. P. (1999). Saccadic performance as a function of the presence and disappearance of auditory and visual fixation stimuli. *J. Cogn. Neurosci.* 11 206–213 10.1162/089892999563337 10198135

[B37] Tenkova T. Young C. Dikranian K. Labruyere J. Olney J. W. (2003). Ethanol-induced apoptosis in the developing visual system during synaptogenesis. *Invest. Ophthalmol. Vis. Sci.* 44 2809–2817 10.1167/iovs.02-0982 12824217

[B38] Uhlhaas P. J. Singer W. (2006). Neural synchrony in brain disorders: relevance for cognitive dysfunctions and pathophysiology. *Neuron* 52 155–168 10.1016/j.neuron.2006.09.020 17015233

[B39] Valenzuela C. F. Morton R. A. Diaz M. R. Topper L. (2012). Does moderate drinking harm the fetal brain? Insights from animal models. *Trends Neurosci.* 35 284–292 10.1016/j.tins.2012.01.006 22402065 PMC3348364

[B40] Van Der Werf J. Buchholz V. N. Jensen O. Medendorp W. P. (2013). Reorganization of oscillatory activity in human parietal cortex during spatial updating. *Cereb. Cortex* 23 508–519 10.1093/cercor/bhr387 22414770

[B41] Van Der Werf J. Jensen O. Fries P. Medendorp W. P. (2008). Gamma-band activity in human posterior parietal cortex encodes the motor goal during delayed prosaccades and antisaccades. *J. Neurosci.* 28 8397–8405 10.1523/JNEUROSCI.0630-08.2008 18716198 PMC6671049

[B42] Zhang M. Barash S. (2004). Persistent LIP activity in memory antisaccades: working memory for a sensorimotor transformation. *J. Neurophysiol.* 91 1424–1441 10.1152/jn.00504.2003 14523076

[B43] Zucca S. Valenzuela C. F. (2010). Low concentrations of alcohol inhibit BDNF-dependent GABAergic plasticity via L-type Ca2+ channel inhibition in developing CA3 hippocampal pyramidal neurons. *J. Neurosci.* 30 6776–6781 10.1523/JNEUROSCI.5405-09.2010 20463239 PMC2878312

